# Zero-Iodinated-Contrast CO_2_-Guided Atherectomy for Superficial Femoral Artery Chronic Total Occlusions in Advanced Chronic Kidney Disease

**DOI:** 10.3390/jcm15218709

**Published:** 2026-11-08

**Authors:** Özgür Zülfükar Ertuğrul, Esma Deniz Barc, Mehmet Cudi Tuncer

**Affiliations:** 1 Department of Radiology, Gazi Yaşargil Training and Research Hospital, University of Health Sciences, Diyarbakır 21090, Turkey; 2 Department of Anatomy, Faculty of Medicine, Dicle University, Diyarbakir 21200, Turkey; drdenizbarc@outlook.com (E.D.B.); drcudi@hotmail.com (M.C.T.)

**Keywords:** chronic kidney disease, peripheral arterial disease, chronic total occlusion, atherectomy, carbon dioxide angiography, contrast-induced nephropathy

## Abstract

**Background/Objectives:** Patients with chronic kidney disease (CKD) who undergo complex femoropopliteal endovascular interventions are at heightened risk of contrast-associated acute kidney injury (CA-AKI). This study assessed technical feasibility, short-term creatinine-based renal outcomes, and clinical outcomes of a zero-iodinated-contrast, carbon dioxide (CO_2_)-guided atherectomy approach for chronic total occlusions (CTOs) of the superficial femoral artery (SFA). **Materials and Methods:** In this retrospective, single-center analysis, 25 non-dialysis patients with CKD underwent CO_2_-only angiography-guided atherectomy for SFA CTOs. Renal function, procedural characteristics, distal embolization, limb-related outcomes, and duplex ultrasound-defined patency were evaluated. Serum creatinine was measured at baseline and within 48 h after the procedure. **Results:** The mean age was 65.4 ± 9.8 years, and 36% of patients presented with chronic limb-threatening ischemia (CLTI). Baseline serum creatinine was 2.16 ± 0.44 mg/dL, and the estimated glomerular filtration rate (eGFR) was 29.5 ± 9.1 mL/min/1.73 m^2^; 56% of patients had Stage 4 CKD. Technical success was achieved in all procedures. Cumulative CO_2_-volume data were available for 24 patients, with a mean volume of 98.8 ± 37.6 mL, and the mean procedure duration was 38.2 ± 17.2 min. Distal embolization occurred in 20% of patients (5/25), including one event requiring catheter-directed thrombolysis; all embolic events were treated endovascularly, with near-complete recanalization in the thrombolysis case. Mean serum creatinine changed by −0.016 ± 0.086 mg/dL, and no creatinine-defined AKI was observed within the available 48-h assessment. No patient required initiation of dialysis. Follow-up outcomes were available for 24 of 25 patients (96%); one patient was lost to follow-up. Over a mean follow-up period of 26 months (with incompletely reconstructed surveillance), no duplex-defined restenosis, recurrent CTO, or target lesion revascularization (TLR) was documented among patients with available follow-up imaging; this is a descriptive observation within the available follow-up and does not establish universal or long-term freedom from restenosis. **Conclusions:** A zero-iodinated-contrast, CO_2_-guided atherectomy strategy appears technically feasible for SFA CTO revascularization in patients with advanced CKD and was associated with observed short-term creatinine stability within the available 48-h assessment in this small retrospective cohort. These preliminary observations require validation in larger, prospective, controlled studies.

## 1. Introduction

CKD is independently associated with an increased prevalence of peripheral arterial disease (PAD) and confers substantial additional cardiovascular and procedural risk [1]. Even modest reductions in eGFR are linked to a graded, dose-dependent elevation in the risk of mortality, cardiovascular events, and hospitalization, with risk rising sharply once eGFR declines below 45 mL/min/1.73 m^2^ [2,3].

PAD itself constitutes a major global health burden, affecting an estimated 200 million individuals worldwide, with diabetes mellitus, cigarette smoking, and arterial hypertension representing the principal modifiable risk factors [4,5]. When CKD and PAD coexist, clinical outcomes are disproportionately adverse: affected patients more frequently present with extensive, heavily calcified, multilevel arterial disease, have higher rates of major adverse limb events, and are more often denied revascularization because of concerns regarding procedure-related renal injury.

Contemporary guidelines for the management of PAD and CLTI increasingly emphasize individualized, risk-stratified revascularization strategies, with particular attention to patients with concomitant CKD, in whom procedural risk is markedly increased [6,7,8,9,10]. However, none of these consensus documents offers detailed technical recommendations on how to safely perform revascularization of complex CTOs in patients with advanced CKD, leaving these decisions largely dependent on operator expertise and institutional practice patterns.

Endovascular revascularization exposes patients with CKD to an additional, procedure-related hazard: contrast-associated AKI (CA-AKI), which is among the most frequent causes of hospital-acquired renal dysfunction [11,12]. Iodinated contrast agents are believed to exert nephrotoxic effects via a combination of direct tubular cytotoxicity, medullary hypoxia resulting from altered renal hemodynamics, and the generation of reactive oxygen species; these effects are substantially amplified when baseline renal reserve is reduced [11,12,13].

Consensus statements from radiology and nephrology societies underscore that patients with an eGFR below 30 mL/min/1.73 m^2^ are at particularly high risk, with reported CA-AKI incidences commonly exceeding 20–30% in this subgroup depending on contrast volume and comorbidity burden. These guidelines also emphasize that periprocedural isotonic fluid administration remains the cornerstone of prevention, as pharmacologic prophylaxis has not consistently mitigated this risk in large randomized clinical trials [13,14]. Importantly, even transient AKI following angiographic procedures has been associated with adverse long-term cardiovascular and renal outcomes, including accelerated progression to end-stage renal disease [15,16], a risk captured by the KDIGO diagnostic criteria [17].

Gadolinium-based contrast agents, once considered a potential alternative to iodinated contrast in patients with impaired renal function, have been implicated in the development of nephrogenic systemic fibrosis and therefore cannot be regarded as a suitable substitute in this population [18].

CO_2_ angiography has been employed for several decades as a renal-sparing alternative to iodinated contrast, enabling diagnostic-quality vascular imaging without nephrotoxic or allergenic potential, as CO_2_ is rapidly absorbed and eliminated via the pulmonary circulation rather than being filtered by the kidneys [19,20]. Early technical refinements addressed issues related to image artifacts and interference from bowel gas, thereby establishing CO_2_ angiography as a reproducible modality even in patients with severe renal insufficiency [20,21].

In the coronary literature, analogous near-zero-contrast strategies utilizing intravascular ultrasound guidance have demonstrated that substantial reductions in iodinated contrast volume can be achieved without compromising procedural success [22], supporting the broader applicability of contrast-minimizing approaches across vascular territories. Nonetheless, despite decades of accumulated experience, the use of strict CO_2_-only imaging in CTO interventions of the femoropopliteal segment among the most technically challenging and contrast-intensive procedures in peripheral vascular practice, given the need for repeated re-entry attempts and precise assessment of debulking outcomes remains sparsely documented.

SFA CTOs typically necessitate prolonged fluoroscopy times, multiple angiographic projections, and substantial contrast volumes to accomplish lesion crossing, true-lumen re-entry, and accurate evaluation of debulking and angioplasty results, all of which may increase the risk of CA-AKI in patients with CKD [12,13]. Published evidence specifically evaluating strict CO_2_-only guidance combined with mechanical atherectomy for SFA CTOs in patients with advanced non-dialysis CKD remains limited.

To address this knowledge gap, we evaluated technical feasibility and short-term creatinine-based renal outcomes of this approach, as well as technical success, procedural complications, duplex ultrasound-defined patency, and mid-term clinical and limb-related outcomes.

## 2. Materials and Methods

### 2.1. Study Design and Population

This retrospective, single-center study included 25 non-dialysis patients with chronic kidney disease (CKD) who had undergone endovascular treatment for isolated superficial femoral artery (SFA) chronic total occlusion (CTO) between May 2022 and March 2024. The study was based on the retrospective evaluation of existing clinical and procedural records, and research-related data extraction and analysis were performed after ethics committee approval.

**Figure 1:**
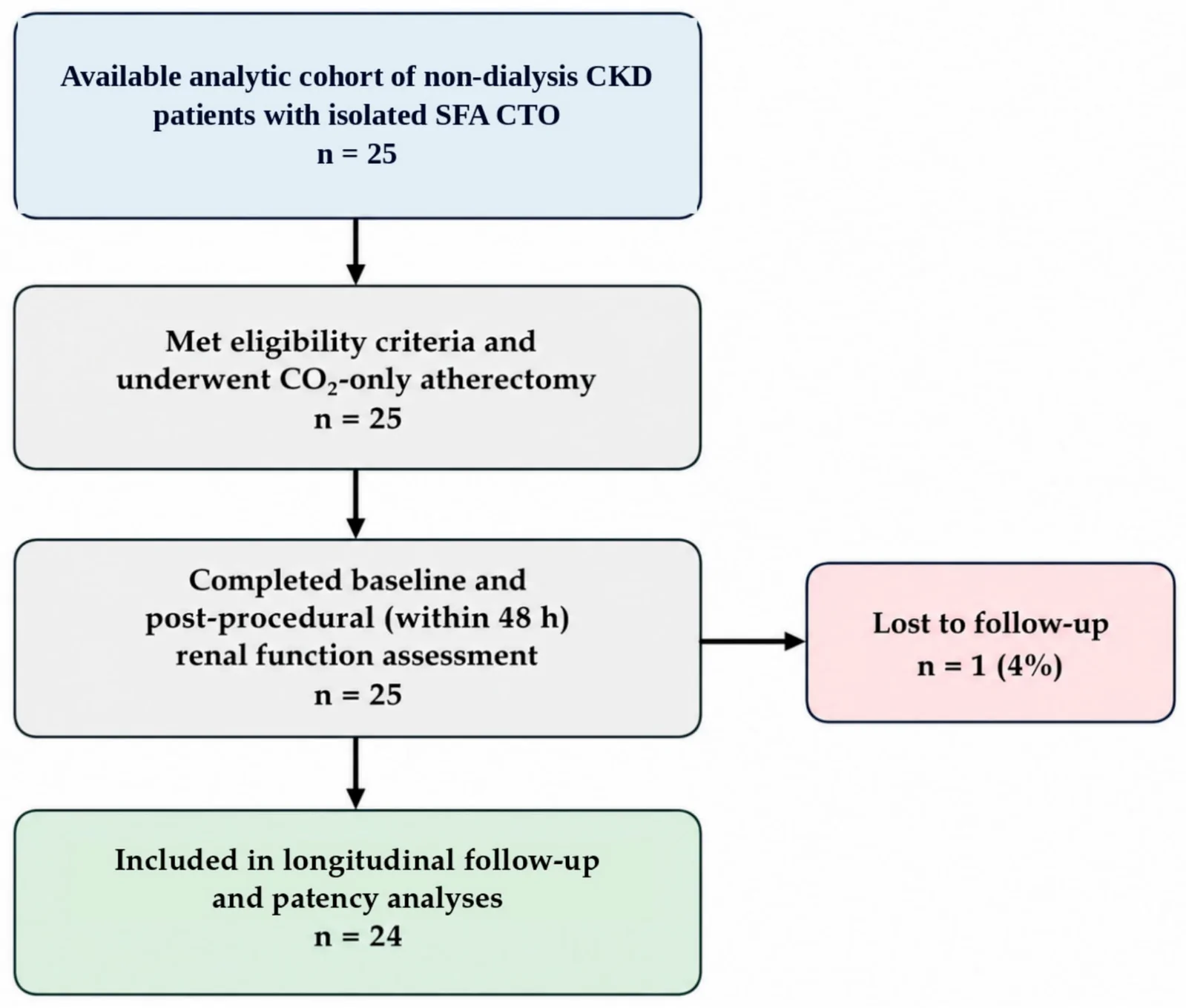
Patient flow diagram. Available analytic cohort: 25 non-dialysis patients with CKD who met the study eligibility criteria and underwent CO_2_-only atherectomy. All 25 patients completed baseline and post-procedural renal function assessment within 48 h. One patient was lost to longitudinal follow-up; therefore, 24 patients were included in the longitudinal follow-up and patency analyses. The complete number of patients screened and excluded before treatment could not be reconstructed from the available retrospective records.

Patients were eligible for inclusion if they had established CKD, an angiographically confirmed SFA CTO requiring endovascular revascularization, and at least two-vessel below-the-knee runoff, as confirmed by duplex ultrasonography. Patients receiving maintenance dialysis were excluded. Patients with lesions requiring treatment of major inflow disease or without adequate distal runoff for the planned revascularization strategy were not included in the study cohort. The patient selection process is summarized in Figure 1.

The available analytic cohort comprised 25 non-dialysis patients with CKD who met the eligibility criteria and underwent CO_2_-only atherectomy; a complete screening log allowing reconstruction of the total number of patients assessed for eligibility and excluded before treatment was not available for this retrospective analysis. Accordingly, the available cohort flow begins with the 25 patients meeting the study inclusion criteria, and this limits assessment of selection bias in the source population. Pre-procedural duplex ultrasonography was used to assess below-the-knee runoff. The SFA CTO was confirmed angiographically at the time of intervention using CO_2_ angiography, which also provided procedural guidance.

The indication for intervention was symptomatic SFA CTO with either intermittent claudication (16 patients, 64%) or chronic limb-threatening ischemia (9 patients, 36%), as detailed in Section 3.1. Details of prior guideline-directed medical and supervised-exercise therapy in patients presenting with claudication, and standardized lesion-calcification grading and detailed plaque morphology, were not systematically available in the retrospective analytic dataset and could therefore not be reported.

The study was conducted in accordance with the principles of the Declaration of Helsinki and was approved by the Clinical Research Ethics Committee of Gazi Yaşargil Training and Research Hospital, University of Health Sciences (approval no. 363; approval date: 14 August 2026). Written informed consent for the interventional procedure had been obtained from the patients in accordance with institutional practice. The institutional consent form included permission for data generated during or as a result of the procedure to be used for scientific research purposes.

The exact data cut-off date for retrospective data extraction and analysis was not recorded in the available analytic dataset.

### 2.2. Renal Assessment

Serum creatinine was measured at baseline and reassessed within the first 48 h after the procedure. eGFR was calculated using the CKD-Epidemiology Collaboration (CKD-EPI) equation [23], and CKD severity was categorized according to the corresponding eGFR-based stages.

Post-procedural AKI was assessed according to the serum creatinine-based KDIGO criterion, defined as an absolute increase in serum creatinine of ≥0.3 mg/dL from baseline within 48 h. Urine output and the KDIGO 7-day relative increase criterion were not systematically recorded in this retrospective cohort and therefore could not be applied. Accordingly, AKI assessment was based exclusively on the available serum creatinine criterion.

### 2.3. Procedural Technique

All interventions were performed in a dedicated angiography suite under mild conscious sedation. Patients were positioned supine with the lower extremities elevated by approximately 15° to facilitate CO_2_ angiographic flow dynamics. Hemodynamic status and oxygen saturation were monitored throughout the procedure.

No iodinated contrast medium was administered during lesion visualization, CTO crossing, atherectomy, adjunctive balloon angioplasty, or completion angiography. Thus, a zero-iodinated-contrast strategy was maintained throughout the intervention.

#### 2.3.1. Vascular Access Strategy

Vascular access was obtained under ultrasound guidance in all cases. The access route was selected according to lesion location and the anticipated crossing strategy. Contralateral retrograde common femoral artery access was preferentially used for proximal SFA CTOs, whereas ipsilateral antegrade common femoral artery access was used for mid- and distal-SFA CTOs.

A 7F long vascular sheath (Terumo, Tokyo, Japan) was used in all patients and continuously flushed with pressurized saline.

When antegrade lesion crossing was unsuccessful or considered technically unfavorable, retrograde popliteal artery access was obtained under ultrasound guidance. In these cases (n = 6), the CTO was crossed retrogradely, and through-and-through wire access was established using a snare technique.

#### 2.3.2. Anticoagulation Protocol

Following sheath insertion, all patients received an initial intravenous bolus of 5000 IU unfractionated heparin. During prolonged procedures, additional unfractionated heparin was administered at 2000 IU/h to maintain procedural anticoagulation.

Details of intra-procedural anticoagulation monitoring (e.g., activated clotting time and target range) and the post-procedural antiplatelet regimen (agent[s] and duration) were not systematically recorded in the analytic dataset.

#### 2.3.3. CO_2_ Angiography Protocol

CO_2_ digital subtraction angiography (DSA) was used as the sole angiographic contrast technique throughout the intervention. CO_2_ was administered in controlled injections according to the vascular territory being evaluated, with sufficient intervals between injections to allow clearance of the gas before subsequent image acquisition. Injection volume and frequency were adjusted according to lesion location, image quality, and procedural requirements while avoiding unnecessary cumulative CO_2_ administration.

DSA acquisitions were obtained after CO_2_ administration for visualization of the target vessel, CTO morphology, distal runoff, and procedural roadmap guidance. Repeat acquisitions were performed when required for lesion crossing, assessment of the treated segment, or confirmation of distal flow. The cumulative CO_2_ volume administered during each procedure was recorded for subsequent analysis.

Particular attention was paid to avoiding air contamination during CO_2_ delivery and to allowing adequate gas clearance between consecutive injections. Iodinated contrast was not used as a rescue imaging agent in any of the included procedures.

Device-specific details of the CO_2_ delivery system (device name and manufacturer), the exact volume administered per individual injection, injection pressure/rate settings, the exact interval between consecutive injections, and structured documentation of CO_2_-specific contraindication screening were not recorded in the analytic dataset.

#### 2.3.4. Lesion Crossing

CTOs were crossed using a hydrophilic guidewire supported by a microcatheter. Crossing was performed under fluoroscopic and CO_2_ angiographic roadmap guidance. True-lumen re-entry and distal intraluminal position were confirmed by CO_2_ angiography and, when angiographic assessment was considered insufficient, adjunctive duplex ultrasonography.

Cohort-level counts of intraluminal versus subintimal crossing, the specific re-entry technique used in each subintimal case, and the exact number of cases in which duplex ultrasonography was required to confirm distal intraluminal position were not separately recorded in the analytic dataset beyond the six patients already reported as requiring retrograde popliteal access.

If antegrade crossing was unsuccessful or technically unfavorable, a retrograde approach was used as described above. Following successful retrograde crossing, through-and-through wire access was established when necessary to facilitate subsequent atherectomy and balloon treatment.

#### 2.3.5. Atherectomy Technique and Device Selection

Following successful lesion crossing, atherectomy was performed as the primary debulking strategy.

Directional atherectomy using the HawkOne device (Medtronic Vascular, Inc., Plymouth, MN, USA) was performed in 10 patients. In these procedures, distal embolic protection was provided using a SpiderFX filter (ev3, Inc., Medtronic, Plymouth, MN, USA) positioned in the distal popliteal segment.

Rotational atherectomy using the Jetstream device was performed in 15 patients. These procedures were performed without distal filter protection because of device-related limitations in the use of distal embolic protection.

Because of the retrospective nature of the study, atherectomy device selection was not based on prospective randomization. Device choice reflected procedural considerations, lesion characteristics, device suitability, and operator judgment. Consequently, comparisons between the two atherectomy device groups were considered exploratory rather than comparative effectiveness analyses.

#### 2.3.6. Jetstream-Specific Embolization Reduction Strategy

To minimize the risk of distal embolization during Jetstream atherectomy, a standardized stepwise modified technique was applied. First, the distal portion of the CTO was localized using duplex ultrasonography, and external skin marking was used as a fluoroscopic reference. Atherectomy was intentionally terminated approximately 1 cm proximal to the distal CTO cap. The cutting blades were then deactivated, and prolonged aspiration was performed to reduce the residual particulate burden. Finally, atherectomy of the remaining distal segment was completed in a controlled manner.

In three cases (n = 3), temporary distal flow control was additionally achieved using popliteal artery balloon occlusion through crural access, providing adjunctive embolic protection.

#### 2.3.7. Adjunctive Drug-Coated Balloon Angioplasty

Following atherectomy, all treated SFA segments underwent prolonged angioplasty using appropriately sized drug-coated balloons (DCBs). Balloon diameter was selected according to the estimated reference vessel diameter, with 5–6 mm balloons typically used in the study cohort. Balloon inflation was maintained for approximately 5 min to optimize luminal gain and minimize elastic recoil.

Completion assessment was performed using CO_2_ angiography. In patients with CLTI, additional below-the-knee angioplasty was performed when clinically relevant stenotic disease requiring treatment was identified.

Routine primary stent implantation was not part of the treatment strategy. Bailout stenting was reserved for lesions with persistent flow-limiting dissection or elastic recoil despite prolonged balloon angioplasty.

The drug-coated balloon manufacturer and antiproliferative drug, the distribution of balloon lengths used, the number of balloons used per lesion, and the exact number and extent of adjunctive below-the-knee interventions were not systematically recorded in the analytic dataset beyond the balloon diameter range and the general indication for below-the-knee angioplasty already reported.

#### 2.3.8. Management of Procedural Complications

Flow-limiting dissections were initially managed using prolonged balloon inflation. Bailout deployment of a self-expanding covered stent graft was reserved for cases with persistent flow limitation or elastic recoil despite balloon angioplasty.

Distal embolization was primarily managed using aspiration thrombectomy with a small-caliber catheter. Catheter-directed thrombolysis was reserved for embolic events refractory to aspiration or involving distal below-the-knee vessels.

### 2.4. Post-Procedural Care

Serum creatinine was reassessed within 48 h after the procedure. Patients without reduced left ventricular function received isotonic saline infusion for 24 h. In three patients with reduced ejection fraction, post-procedural hydration was withheld to minimize the risk of volume overload.

Clinical and vascular examinations were performed after the intervention, with particular attention to access-site complications, distal perfusion, and procedure-related adverse events. Patients were typically discharged approximately 48 h after the procedure when clinically stable.

### 2.5. Follow-Up and Outcome Assessment

Clinical and duplex Doppler ultrasonographic follow-up was scheduled at 1 month, 6 months, and every 6 months thereafter.

Duplex ultrasonography was used as the primary imaging modality for post-procedural surveillance. Restenosis was assessed using changes in peak systolic velocity and peak systolic velocity ratios across the treated segment according to established duplex criteria [24]. Hemodynamically significant restenosis was defined as ≥50% diameter stenosis. Primary patency was defined as freedom from duplex-defined ≥50% restenosis or TLR during follow-up.

Clinical follow-up included assessment of recurrent or persistent symptoms, including intermittent claudication and CLTI-related manifestations. In patients presenting with CLTI, wound status and the occurrence of minor or major amputation were recorded during follow-up. Limb salvage was defined by freedom from major amputation of the treated limb. At the routine 1- and 6-month follow-up visits, patients who had presented with intermittent claudication were also asked about changes in claudication symptoms. This was a subjective, patient-reported clinical assessment based on whether walking distance had increased and exertional leg pain had decreased compared with the pre-procedural status. No standardized treadmill test or formal walking-distance measurement was performed.

The independence or blinding status of the duplex ultrasound assessor relative to the treating/interventional team could not be established from the available records. Baseline and follow-up ankle–brachial index, serial Rutherford-category change, WIfI stage, and time to wound healing were not systematically recorded in the analytic dataset; clinical outcomes are therefore reported using the documented symptom status, wound status, amputation, and TLR data presented in Section 3.5.

### 2.6. Study Endpoints

The primary renal endpoint was the incidence of creatinine-defined AKI within the available 48-h assessment window, according to the serum creatinine-based KDIGO criterion described above.

Secondary endpoints included technical success, procedural complications, primary patency, and clinical outcomes. Technical success was defined as successful CO_2_-only lesion crossing and atherectomy followed by completion of any required adjunctive or bailout treatment, resulting in final residual diameter stenosis < 30% and no residual flow-limiting dissection.

Procedural safety outcomes included distal embolization and dissection. Primary patency was defined as freedom from duplex-defined ≥50% restenosis or TLR during follow-up. Clinical outcomes included symptomatic improvement, wound healing, minor and major amputation, and limb salvage.

Given the retrospective, single-center design, these endpoints were defined for the purposes of the present analysis before data extraction rather than prospectively registered in a trial protocol.

### 2.7. Statistical Analysis

Continuous variables are presented as the mean ± standard deviation (SD). Between-group comparisons were performed using the independent-samples *t*-test or Mann–Whitney U test, as appropriate according to the distribution and characteristics of the data. Categorical variables are presented as frequencies and percentages and were compared using the chi-square test or Fisher’s exact test when expected cell counts were small. Baseline and post-procedural serum creatinine measurements were compared using the paired-samples *t*-test.

Exploratory subgroup analyses were performed according to atherectomy strategy (directional HawkOne vs. rotational Jetstream) for selected procedural and safety outcomes. Correlations between CTO length and continuous procedural parameters, including procedure duration and cumulative CO_2_ volume, were evaluated using Pearson correlation analysis.

All statistical tests were two-sided, and a *p*-value < 0.05 was considered statistically significant. Given the retrospective, single-center design, small overall sample size, and limited subgroup sizes, subgroup and correlation analyses were considered exploratory and hypothesis-generating rather than confirmatory. Statistical analyses were performed using SPSS Statistics version 32.0 (IBM Corp., Armonk, NY, USA).

## 3. Results

### 3.1. Baseline Patient and Lesion Characteristics

A total of 25 non-dialysis patients with CKD and isolated SFA CTO were included in the study (Figure 1). The mean age was 65.4 ± 9.8 years, and 15 patients (60%) were male. Nine patients (36%) presented with CLTI, whereas 16 (64%) had intermittent claudication.

Baseline renal function reflected advanced CKD, with a mean serum creatinine concentration of 2.16 ± 0.44 mg/dL and a mean eGFR of 29.5 ± 9.1 mL/min/1.73 m^2^. Fourteen patients (56%) had Stage 4 CKD, 10 (40%) had Stage 3b CKD, and one (4%) had Stage 3a CKD.

The cohort had a high prevalence of cardiovascular risk factors and comorbidities, including diabetes mellitus in 18 patients (72%), coronary artery disease in 19 (76%), hypertension in 17 (68%), and dyslipidemia in 19 (76%). The mean CTO length was 9.0 ± 3.0 cm (range, 5–14 cm), and the mid one-third of the SFA was the most common lesion location (56%). Baseline demographic, clinical, renal, and lesion characteristics are summarized in Table 1.

### 3.2. Procedural Characteristics and Technical Outcomes

Ipsilateral antegrade access was used in 21 patients (84%), whereas contralateral access was used in four (16%). Retrograde popliteal access was required in six patients (24%) to facilitate lesion crossing. Directional atherectomy with HawkOne was performed in 10 patients (40%), whereas rotational atherectomy with Jetstream was performed in 15 (60%).

Cumulative CO_2_-volume data were available for 24 of 25 patients. The mean cumulative CO_2_ volume among patients with available data was 98.8 ± 37.6 mL, and the mean procedure duration was 38.2 ± 17.2 min. technical success was achieved in all 25 patients (100%).

CTO length was significantly correlated with procedure duration (Pearson’s r = 0.43, *p* = 0.030) and cumulative CO_2_ volume (r = 0.58, *p* = 0.003).

Exploratory analysis according to atherectomy device showed a mean procedure duration of 41.5 ± 21.0 min with Jetstream and 33.2 ± 7.1 min with HawkOne (*p* = 0.25). Among patients with available CO_2_-volume data, the mean CO_2_ volume was 106.7 ± 44.5 mL in the Jetstream group (n = 15) and 85.6 ± 16.7 mL in the HawkOne group (n = 9), respectively (*p* = 0.19). One HawkOne patient had no recorded cumulative CO_2_-volume value. Distal embolization occurred in 3/15 patients (20%) treated with Jetstream and 2/10 (20%) treated with HawkOne (*p* = 1.00), while dissection occurred in 20% of patients in each group (*p* = 1.00).

### 3.3. Procedural Complications

Distal embolization occurred in five patients (20%). Four embolic events involved proximal below-the-knee vessels and were treated with aspiration thrombectomy using a 5F catheter. One embolic event involving the posterior tibial artery was treated with catheter-directed thrombolysis with tissue plasminogen activator, with near-complete recanalization on completion angiography. This 20% rate is clinically relevant. Event-level information concerning the exact timing of detection, the atherectomy device used in each event, SpiderFX or balloon-occlusion status at the time of each event, hospitalization duration, thrombolysis-related bleeding, subsequent intervention, and other clinical consequences were not systematically available for complete retrospective ascertainment. The efficacy of the embolization-mitigation strategy described in Section 2.3.6 in reducing this rate relative to an unprotected approach has not been directly tested in this study and should not be inferred from these descriptive data alone, as no comparator group was available.

Dissection occurred in five patients (20%). One patient (4%) developed a flow-limiting dissection requiring bailout treatment with a self-expanding covered stent graft. The remaining four patients (16%) had non-flow-limiting dissections that were managed with prolonged balloon angioplasty.

The adverse events systematically documented and reported for this cohort are distal embolization, dissection, bailout covered stent placement, minor amputation, major amputation among patients with available follow-up, and dialysis initiation, as detailed above and in Table 2. Access-site complications, arterial perforation, bleeding unrelated to thrombolysis, CO_2_-related symptoms, mortality, major adverse limb events other than amputation, acute thrombosis, urgent surgical intervention, and rehospitalization were not systematically captured as structured dataset variables and could not be completely ascertained for this retrospective analysis; their absence is therefore not reported as a zero rate.

### 3.4. Renal Outcomes

Mean serum creatinine was 2.16 ± 0.44 mg/dL at baseline and 2.15 ± 0.40 mg/dL after the procedure, corresponding to a mean change of −0.016 ± 0.086 mg/dL. No patient met the serum creatinine-based KDIGO criterion for post-procedural AKI, and no patient required initiation of dialysis. The paired samples *t*-test for this within-patient change was not statistically significant (t(24) = −0.93, *p* = 0.36; 95% CI of the mean change, −0.052 to +0.020 mg/dL). The exact two-sided 95% confidence interval (Clopper–Pearson) for the observed 0/25 creatinine-defined AKI rate was 0–13.7%. Creatinine was measured within 48 h after the procedure; the exact within-window sampling-hour distribution was not available in the analytic dataset. No creatinine-defined AKI was observed within the available 48-h assessment; however, the retrospective design, incomplete KDIGO ascertainment (urine output and the 7-day relative-increase criterion were not available), single-timepoint post-procedural sampling, and absence of a comparator group preclude causal inference regarding renoprotection, and these findings should be interpreted as observed short-term creatinine stability rather than proof of a renal-protective effect.

**Figure 2:**
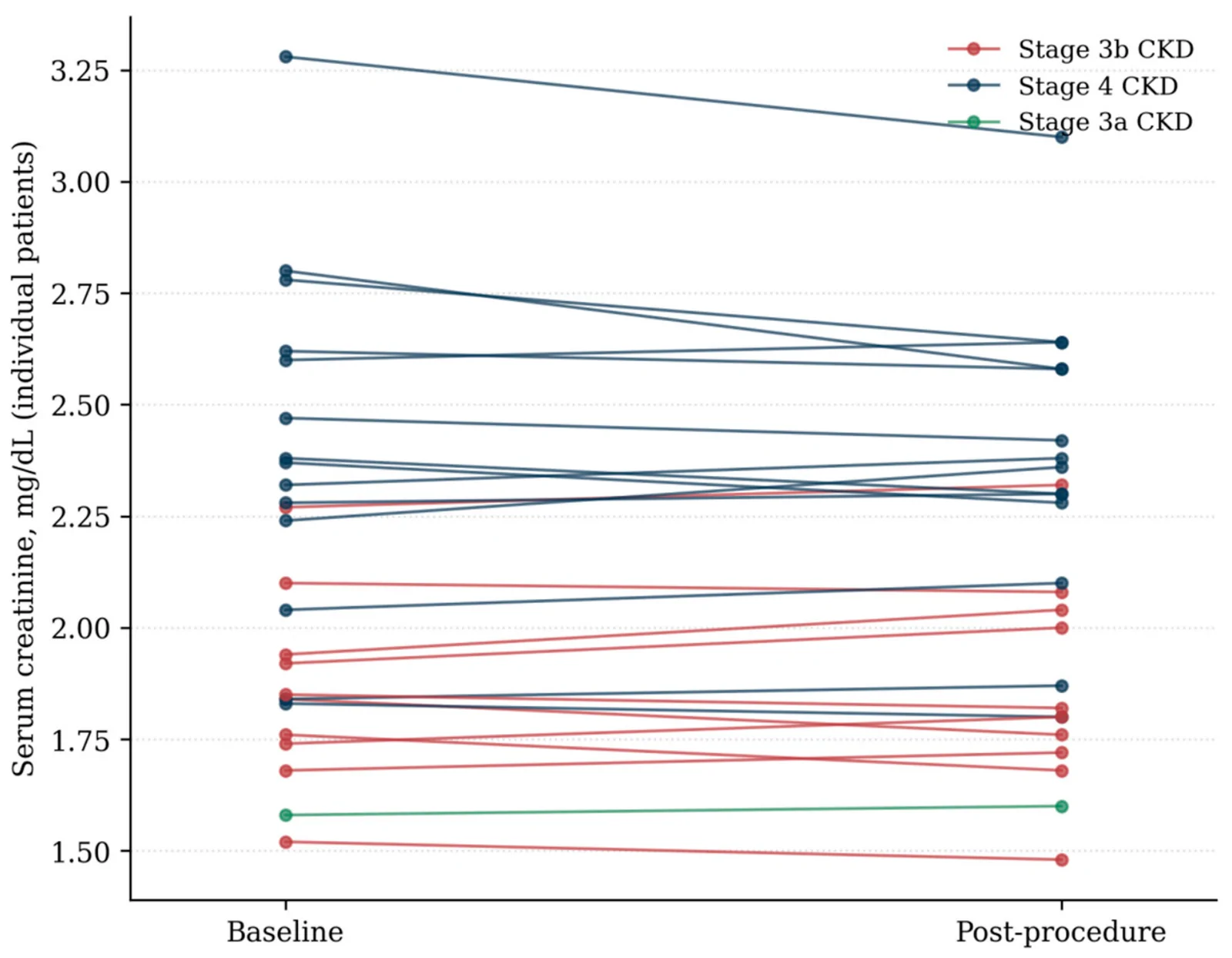
Individual patient serum creatinine trajectories from baseline to post-procedure within 48 h, stratified by baseline CKD stage (Stage 4, Stage 3b, and Stage 3a). No patient met the serum creatinine-based KDIGO criterion for AKI (increase of ≥0.3 mg/dL from baseline).

When stratified according to baseline CKD severity, Stage 4 CKD patients had a mean baseline eGFR of 22.8 ± 4.4 mL/min/1.73 m^2^ (n = 14) and a mean serum creatinine change of −0.036 ± 0.099 mg/dL. Patients with Stage 3b CKD had a mean baseline eGFR of 37.0 ± 4.7 mL/min/1.73 m^2^ (n = 10) and a mean serum creatinine change of +0.008 ± 0.066 mg/dL. The difference in serum creatinine change between these groups was not statistically significant (*p* = 0.24). Individual paired baseline and post-procedural serum creatinine values, stratified by baseline CKD stage, are shown in Figure 2.

The single patient with Stage 3a CKD had a baseline eGFR of 48.2 mL/min/1.73 m^2^ and a serum creatinine change of +0.02 mg/dL and was excluded from the Stage 4 versus Stage 3b comparison. No AKI events occurred in any CKD-stage subgroup.

Among the three patients in whom post-procedural hydration was withheld because of reduced ejection fraction, no post-procedural AKI event was observed.

### 3.5. Clinical and Follow-Up Outcomes

Among the nine patients with CLTI, six (67%) achieved complete wound healing during follow-up, whereas three (33%) underwent minor amputation at the metatarsal level because of progressive infection despite successful revascularization and adjunctive treatment. No major amputations occurred among the patients with available follow-up. Limb salvage among the 24 patients with available follow-up was therefore 24/24 (100%); the patient lost to follow-up was excluded from this denominator. At the routine 1- and 6-month follow-up assessments, all 16 patients who had presented with intermittent claudication reported subjective symptomatic improvement, characterized by an increased self-reported walking distance and/or reduced exertional leg pain compared with baseline.

Follow-up data were available for 24 of 25 patients (96%), with one patient (4%) lost to follow-up. In the source dataset, the patient lost to longitudinal follow-up was not in the CLTI subgroup (CLI negative); given the mutually exclusive presentation categories used in this study (9 CLTI and 16 intermittent claudication), this patient corresponds to the non-CLTI/intermittent-claudication group. All nine patients with CLTI had recorded longitudinal follow-up. Among the 24 patients with available follow-up, four (17%) were followed for 12 months, 12 (50%) for 24 months, and eight (33%) for 36 months. The mean follow-up duration was 26.0 ± 8.4 months (range, 12–36 months).

The exact data cut-off date was not recorded in the available analytic dataset, and the reported follow-up duration for each patient represents the documented available surveillance period rather than a fixed, uniformly applied censoring rule. Interval-level adherence to every individually scheduled duplex examination could not be reconstructed retrospectively for each of the 24 patients with available follow-up, which limits assessment of surveillance completeness.

No duplex-defined ≥50% restenosis was detected among the 24 patients with available follow-up. No recurrent CTO or TLR was recorded during follow-up. Accordingly, primary patency among patients with available follow-up was 24/24 (100%). This is a crude observed proportion among the 24 patients with available follow-up, not a time-to-event estimate; the exact two-sided 95% confidence interval (Clopper–Pearson) for this crude proportion is 85.8–100%. This confidence interval describes the observed proportion only and does not substitute for a time-to-event (Kaplan–Meier) analysis with proper censoring. Patient-level follow-up and censoring dates, duplex completion and numbers at risk at each scheduled interval, and patient-level PSV and PSVR measurements were not available in the analytic dataset, so a time-to-event analysis could not be performed. Because interval-level surveillance completeness could not be reconstructed, the 24/24 result is reported only as a crude observed proportion among patients with available follow-up and may be affected by incomplete surveillance.

### 3.6. Representative Case

**Figure 3:**
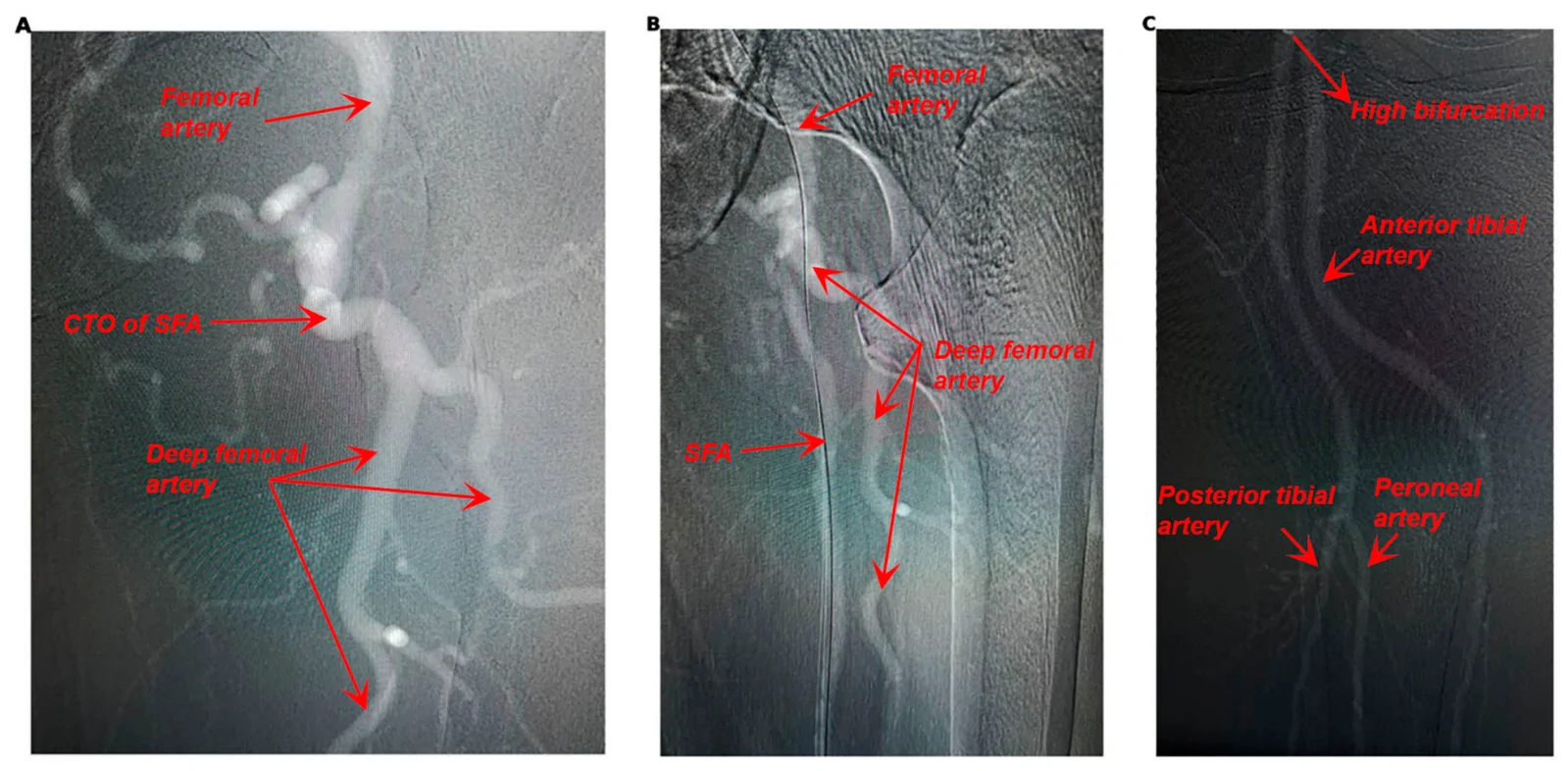
Representative CO_2_-guided endovascular treatment of SFA CTO. (**A**) Baseline CO_2_ angiography demonstrating CTO of the proximal and middle SFA. (**B**) Final CO_2_ angiography following successful CTO crossing, atherectomy, and prolonged DCB angioplasty, showing restoration of antegrade flow without significant residual stenosis. (**C**) Completion CO_2_ angiography demonstrating preserved distal runoff through the anterior tibial, posterior tibial, and peroneal arteries. CO_2_, carbon dioxide; CTO, chronic total occlusion; SFA, superficial femoral artery.

A representative case illustrating the CO_2_-guided treatment strategy is shown in Figure 3. Baseline CO_2_ angiography demonstrated a CTO involving the proximal and middle thirds of the left SFA (Figure 3A). Following successful guidewire crossing, atherectomy and prolonged DCB angioplasty were performed. Final CO_2_ angiography demonstrated successful recanalization with restoration of antegrade flow and no significant residual stenosis (Figure 3B). Completion CO_2_ angiography demonstrated a patent popliteal artery with high trifurcation and preserved distal three-vessel runoff (Figure 3C).

## 4. Discussion

This study demonstrates the technical feasibility of performing complex SFA CTO interventions without iodinated contrast agents in patients with advanced CKD. Despite a high-risk cohort—with more than half of the patients classified as having Stage 4 CKD and a high prevalence of diabetes mellitus, coronary artery disease, hypertension, and dyslipidemia [1,4,5]—technical success was achieved in all 25 procedures under CO_2_-only angiographic guidance, with a mean CO_2_ volume of 98.8 ± 37.6 mL (among the 24 patients with available CO_2_-volume data) and a mean procedure duration of 38.2 ± 17.2 min. These findings should be regarded as preliminary given the retrospective, single-center design, limited sample size, and absence of a conventional contrast-based control group.

CO_2_ angiography has been recognized for several decades as a renal-sparing imaging modality [19,20]. In contrast to iodinated or gadolinium-based contrast agents, CO_2_ is eliminated via the pulmonary circulation rather than the kidneys and lacks direct tubular cytotoxicity, providing a mechanistic rationale for avoiding iodinated contrast exposure; however, the present study assessed only short-term creatinine-based outcomes and did not evaluate renal histology or other markers of tubular injury. Nevertheless, the adoption of CO_2_ angiography in complex peripheral interventions, particularly CTO lesions, has been limited owing to concerns regarding image quality and procedural feasibility. More recent studies have demonstrated the safety and efficacy of CO_2_ angiography in PAD [21], but evidence specifically addressing CTO interventions remains scarce, and published evidence specifically evaluating strict CO_2_-only guidance combined with mechanical atherectomy for SFA CTOs in patients with advanced non-dialysis CKD remains limited. In the present cohort, all procedures were completed successfully using CO_2_-only imaging, supporting the procedural feasibility of this approach even in technically challenging lesions and aligning with near-zero-contrast strategies described in the coronary literature [22].

Laser atherectomy represents a complementary recanalization and plaque-modification option that may merit consideration alongside the CO_2_-guided, mechanical-atherectomy approach described here, particularly in heavily calcified femoropopliteal disease, which is frequently encountered in advanced CKD. Balloon-assisted excimer-laser atherectomy via a transpopliteal approach has been described as a feasible bail-out technique for refractory femoropopliteal CTOs after failed antegrade crossing [25], and more recently a 355 nm laser atherectomy system has shown encouraging technical success and 12-month outcomes in femoropopliteal in-stent occlusions compared with conventional recanalization [26]. Laser-assisted techniques may facilitate progressive intraluminal channel creation and plaque debulking, potentially reducing the need for extensive subintimal recanalization and re-entry manoeuvres in selected lesions. This possibility is presented here as a complementary option rather than as evidence of superiority; the present study did not evaluate laser atherectomy, and dialysis-dependent patients, in whom heavily calcified disease is most prevalent, were excluded from this cohort.

A key technical aspect of this study is the standardized application of an embolization-mitigation strategy, particularly in cases involving Jetstream atherectomy. Distal embolization is a well-recognized complication of atherectomy, with reported incidence rates ranging from 10% to 25%, depending on lesion complexity, calcific burden, and the use of embolic protection devices [27,28]. Distal embolization occurred in five patients (20%), and all five events received endovascular treatment, including catheter-directed thrombolysis in one patient. HawkOne directional atherectomy [29,30] was performed in 10 patients, all with SpiderFX distal filter protection, whereas Jetstream rotational atherectomy [31] was performed in 15 patients without distal filter protection because of device-related limitations; temporary popliteal balloon occlusion through crural access was used selectively in three cases. Distal embolization occurred in 2 of 10 HawkOne patients and 3 of 15 Jetstream patients, an observed crude rate of 20% in both groups (*p* = 1.00). These equal observed proportions do not establish equivalence and do not demonstrate that the two embolic-protection strategies had the same effectiveness: device and protection-strategy selection were not randomized, the groups were small, and confounding by indication and underlying lesion characteristics is likely. Event-level linkage between each embolization event and SpiderFX or temporary balloon-occlusion status was not systematically available for complete retrospective ascertainment, so this study cannot determine the comparative effectiveness of the different embolic-protection approaches; neither the modified Jetstream technique nor temporary balloon occlusion can be inferred to have reduced embolization from these descriptive data alone, as no comparator group was available. CTO length was significantly correlated with both procedural time (r = 0.43, *p* = 0.030) and cumulative CO_2_ volume (r = 0.58, *p* = 0.003), reflecting an association between lesion complexity and procedural burden that should not be interpreted as evidence regarding embolic risk. Ultrasound-guided vascular access, employed in all cases, and retrograde popliteal access, required in six patients when antegrade crossing was unfavorable, are established bail-out strategies for refractory SFA CTOs [32,33] and may be particularly relevant in Jetstream cases, where distal filter protection is unavailable.

No patient met the serum creatinine-based KDIGO criterion for AKI within the available 48-h assessment [17], and no patient required initiation of dialysis. Mean serum creatinine change was −0.016 ± 0.086 mg/dL (paired-samples *t*-test t(24) = −0.93, *p* = 0.36; 95% CI −0.052 to +0.020 mg/dL), and the exact two-sided 95% confidence interval (Clopper–Pearson) for the observed 0/25 creatinine-defined AKI rate was 0–13.7%. This pattern held across CKD-severity subgroups Stage 4 (eGFR 22.8 ± 4.4 mL/min/1.73 m^2^) versus Stage 3b (eGFR 37.0 ± 4.7 mL/min/1.73 m^2^), *p* = 0.24 and in the three patients who did not receive post-procedural hydration because of reduced left ventricular ejection fraction. Creatinine was measured within 48 h after the procedure, but the exact within-window sampling-hour distribution was not available in the analytic dataset; urine output and the 7-day relative-increase KDIGO criterion were not available, renal function was assessed at a single post-procedural time point rather than through serial measurements, and no comparator group was available. These factors preclude causal inference regarding renoprotection, and the renal findings should be interpreted as observed short-term creatinine stability rather than proof of a causal renal-protective effect or evidence that the strategy abolishes AKI risk.

Primary patency among the 24 patients with available follow-up was 24/24 (100%); this is a crude observed proportion, not a time-to-event estimate, and its exact two-sided 95% confidence interval (Clopper–Pearson) is 85.8–100%. Patient-level follow-up and censoring dates, duplex completion and numbers at risk at each scheduled interval, and patient-level PSV and PSVR measurements were not available in the analytic dataset, so a time-to-event (Kaplan–Meier) analysis could not be performed. Because interval-level surveillance completeness could not be reconstructed, this 24/24 result is reported only as a crude observed proportion among patients with available follow-up and may be affected by incomplete surveillance. This crude proportion should therefore be interpreted as a preliminary observation rather than as evidence of long-term durability comparable to that reported in larger controlled trials such as IN.PACT SFA [34]. All 16 patients presenting with intermittent claudication reported symptomatic improvement, and among the nine patients with CLTI, six (67%) achieved complete wound healing while three (33%) underwent minor amputation at the metatarsal level because of progressive infection despite successful revascularization, consistent with wound-healing trajectories described in contemporary CLTI guidelines [7]. No major amputations occurred among the 24 patients with available follow-up, corresponding to a limb salvage rate of 24/24 (100%) in this subgroup; the patient lost to follow-up was excluded from this denominator. The independence or blinding status of the duplex ultrasound assessor relative to the treating team could not be established from the available records, and baseline/follow-up ankle–brachial index, serial Rutherford-category change, WIfI stage, and time to wound healing were not systematically recorded; clinical improvement is therefore reported using the documented symptom, wound, amputation, and TLR outcomes above rather than these more objective measures.

The clinical indication for revascularization in patients presenting with intermittent claudication warrants careful consideration. Intermittent claudication encompasses a broad spectrum of symptom severity and functional impairment, and its presence alone does not constitute a sufficient indication for invasive treatment. Both the 2024 ACC/AHA and European Society of Cardiology (ESC) guidelines emphasize guideline-directed medical therapy, cardiovascular risk-factor modification, and structured or supervised exercise therapy as fundamental components of initial management [35,36]. The ACC/AHA guidelines recommend considering revascularization in patients with functionally limiting claudication who have an inadequate response to guideline-directed medical therapy, including structured exercise, while discouraging intervention in patients who respond adequately to conservative treatment [35]. Similarly, the ESC guidelines recommend reassessing peripheral artery disease-related quality of life after approximately three months of optimal medical treatment and exercise therapy, with revascularization considered selectively when clinically meaningful impairment persists [36]. Supporting this conservative-first approach, a systematic review and meta-analysis of nine randomized controlled trials involving 1477 patients demonstrated no significant differences between invasive and noninvasive treatment in progression to chronic limb-threatening ischemia, major amputation, or all-cause mortality over a mean follow-up of 3.6 years. However, invasive treatment was associated with a significantly higher incidence of subsequent revascularization procedures (RR = 4.15; 95% CI, 2.80–6.16), reinforcing the importance of careful patient selection [37].

In the present study, 16 patients (64%) presented with intermittent claudication, whereas nine (36%) had chronic limb-threatening ischemia, representing distinct clinical presentations and treatment considerations. Although revascularization may be appropriate for selected patients with substantial functional limitations, standardized baseline walking distances, detailed assessments of claudication severity, and prior guideline-directed medical and supervised exercise therapy were not systematically documented in our retrospective dataset. Consequently, the appropriateness of revascularization in individual patients presenting with claudication cannot be independently established from the available records. Furthermore, improvements in walking distance and exertional pain were based on subjective patient assessments rather than standardized functional testing. Therefore, our findings should not be interpreted as demonstrating the superiority of endovascular intervention over conservative management or supporting routine revascularization for intermittent claudication. Instead, this study specifically evaluates the technical feasibility and short-term renal safety of zero-iodinated-contrast CO_2_-guided atherectomy in patients with advanced chronic kidney disease undergoing intervention for superficial femoral artery chronic total occlusions.

This study has several important limitations. The retrospective, single-center design and small sample size reduce statistical power and restrict external validity, and the absence of a contemporaneous comparator cohort undergoing conventional iodinated contrast-guided intervention precludes direct comparison of renal, procedural, and clinical outcomes and attribution of the absence of post-procedural AKI solely to the zero-iodinated-contrast strategy. A complete screening log allowing reconstruction of the total number of patients assessed and excluded before treatment was not available for this retrospective analysis; the available cohort flow begins with the 25 patients meeting the study inclusion criteria, which limits assessment of selection bias in the source population. Lesion complexity could not be reported using a reliable TASC II classification, because the original classification was inconsistent with standard lesion-length criteria and the patient-level lesion morphology required for valid reclassification was not available; TASC II categories and any associated subgroup analysis have accordingly been omitted from this report. Several procedural details including prior guideline-directed medical and supervised-exercise therapy, standardized lesion-calcification grading, device-specific CO_2_ delivery settings, cohort-level intraluminal-versus-subintimal crossing and re-entry-technique counts, DCB manufacturer and balloon-length distribution, the exact extent of adjunctive below-the-knee intervention, anticoagulation-monitoring details, and the post-procedural antiplatelet regimen were not systematically recorded in the analytic dataset. Renal function was evaluated using a single post-procedural creatinine measurement within 48 h rather than serial measurements at predefined intervals, and AKI ascertainment relied exclusively on the serum creatinine-based KDIGO criterion, as urine output data and the 7-day relative-increase criterion were not systematically available, so delayed AKI events or episodes defined solely by urine output changes may have gone undetected. Follow-up data were missing for one patient; interval-level adherence to every scheduled duplex examination could not be reconstructed retrospectively, and valid patient-level follow-up and censoring dates were not available, precluding a time-to-event analysis of patency. Objective measures of clinical status baseline/follow-up ABI, serial Rutherford category, WIfI stage, and time to wound healing were not systematically available, and event-level detail for distal embolization (timing, device, protection status, hospitalization, and subsequent intervention) was incompletely ascertainable. All procedures were performed by operators with substantial expertise in CO_2_-guided imaging and complex CTO intervention, and atherectomy device selection was non-randomized with relatively small HawkOne and Jetstream subgroups; consequently, the high technical success rate and device-specific findings may not be generalizable to centers with less experience or to broader patient populations. Larger prospective, multicenter, controlled trials incorporating standardized CO_2_ imaging protocols, complete source-population and procedural documentation, serial renal function assessment, objective functional endpoints, and extended vascular surveillance with valid time-to-event data are warranted to more definitively evaluate the renal safety, procedural feasibility, and long-term durability of this therapeutic approach.

Taken together, these observations indicate that procedural risk in patients with CKD and complex femoropopliteal disease may potentially be addressed not only through minimization of contrast dose but also, in selected cases, through complete avoidance of iodinated contrast, by integrating CO_2_-only angiographic guidance with atherectomy, DCB angioplasty, and targeted measures to reduce and manage distal embolization. The relevance of this approach lies not in any single procedural element but in the demonstration that these components can be combined into a fully iodinated-contrast-free workflow for SFA CTO revascularization. Given the limitations above, this strategy should be regarded as a feasibility signal in a small, highly selected cohort rather than as established practice for patients with advanced CKD undergoing SFA CTO revascularization.

## 5. Conclusions

This retrospective, single-center study indicates that a strictly zero-iodinated-contrast, CO_2_-guided atherectomy strategy was technically feasible for the endovascular management of SFA CTOs in this small, highly selected cohort of patients with advanced CKD, with technical success achieved in all 25 procedures. The approach was associated with observed short-term creatinine stability within the available 48-h assessment, without evidence of a causal renoprotective effect. Distal embolization occurred in 20% of patients (5/25), including one event requiring catheter-directed thrombolysis. The crude patency finding of 24/24 among patients with available follow-up, and the accompanying clinical outcomes, are limited by the small sample size, variable follow-up duration, incomplete reconstruction of surveillance completeness, and the absence of a comparator group, and should not be interpreted as evidence of durable or comparatively effective patency. Prospective, controlled, multicenter validation is required before this strategy can be considered an established alternative to conventional contrast-based revascularization in this population.

## Figures and Tables

**Table 1:** Baseline demographic, comorbidity, and lesion characteristics of the study cohort (n = 25).

Characteristic	Value
Age, years, mean ± SD	65.4 ± 9.8
Male sex, n (%)	15 (60)
Diabetes mellitus, n (%)	18 (72)
Coronary artery disease, n (%)	19 (76)
Hypertension, n (%)	17 (68)
Dyslipidemia, n (%)	19 (76)
Current smoking, n (%)	9 (36)
Chronic limb-threatening ischemia, n (%)	9 (36)
Stage 4 CKD (eGFR 15–29 mL/min/1.73 m^2^), n (%)	14 (56)
Stage 3b CKD (eGFR 30–44 mL/min/1.73 m^2^), n (%)	10 (40)
Stage 3a CKD (eGFR 45–59 mL/min/1.73 m^2^), n (%)	1 (4)
CTO length, cm, mean ± SD (range)	9.0 ± 3.0 (5–14)
Mid one-third SFA lesion, n (%)	14 (56)
Distal one-third SFA lesion, n (%)	7 (28)
Proximal one-third SFA lesion, n (%)	4 (16)

CKD, chronic kidney disease; eGFR, estimated glomerular filtration rate; CTO, chronic total occlusion; SFA, superficial femoral artery; SD, standard deviation.

**Table 2:** Procedural, renal, and clinical outcomes of the study cohort.

Outcome	Value
Technical success, n (%)	25 (100)
Distal embolization, n (%)	5 (20)
Any dissection, n (%)	5 (20)
Flow-limiting dissection, n (%)	1 (4)
Non-flow-limiting dissection, n (%)	4 (16)
Bailout covered stent graft, n (%)	1 (4)
Baseline serum creatinine, mg/dL, mean ± SD	2.16 ± 0.44
Post-procedural serum creatinine, mg/dL, mean ± SD	2.15 ± 0.40
Δ Serum creatinine, mg/dL, mean ± SD	−0.016 ± 0.086; 95% CI −0.052 to +0.020; paired *t*-test *p* = 0.36
Creatinine-defined AKI within the available 48-h assessment, n (%)	0 (0); 95% CI 0–13.7%
Dialysis initiation, n (%)	0 (0)
Follow-up duration, months, mean ± SD (range)	26.0 ± 8.4 (12–36)
Lost to follow-up, n (%)	1 (4)
Primary patency among patients with follow-up, n/N (%)	24/24 (100); 95% CI 85.8–100%
Symptomatic improvement, claudication subgroup, n/N (%)	16/16 (100)
Complete wound healing, CLTI subgroup, n/N (%)	6/9 (67)
Minor amputation, CLTI subgroup, n/N (%)	3/9 (33)
Major amputation, n (%)	0 (0)
Limb salvage among patients with available follow-up, n/N (%)	24/24 (100)

***Note:*** One patient was lost to follow-up and was excluded from the patency analysis; therefore, primary patency was calculated in 24 patients with available follow-up. Other outcomes are reported using the applicable study or subgroup denominator. Limb salvage is likewise reported as 24/24 (100%) among patients with available follow-up, rather than 25/25; the patient lost to follow-up was excluded from the limb-salvage denominator. Symptomatic improvement was based on patient-reported reduction in claudication symptoms at the routine 1- and 6-month follow-up assessments.

## Data Availability

The data presented in this study is available on request from the corresponding author.

## Abbreviations

AKI	Acute kidney injury
CA-AKI	Contrast-associated acute kidney injury
CKD	Chronic kidney disease
CKD-EPI	Chronic Kidney Disease Epidemiology Collaboration
CLTI	Chronic limb-threatening ischemia
CO_2_	Carbon dioxide
CTO	Chronic total occlusion
DCB	Drug-coated balloon
DSA	Digital subtraction angiography
eGFR	Estimated glomerular filtration rate
KDIGO	Kidney Disease: Improving Global Outcomes
PAD	Peripheral arterial disease
SD	Standard deviation
SFA	Superficial femoral artery
TLR	Target lesion revascularization
